# Editorial: Thyroid eye disease

**DOI:** 10.3389/fopht.2026.1780311

**Published:** 2026-01-30

**Authors:** Alon Kahana, Raymond I. Cho, Tarjani V. Dave, Francesco M. Quaranta-Leoni, Diego Strianese

**Affiliations:** 1 Kahana Oculoplastic and Orbital Surgery, Livonia, MI, United States; 2 Department of Ophthalmology, Oakland University William Beaumont School of Medicine, Royal Oak, MI, United States; 3 Department of Ophthalmology and Visual Sciences, The Ohio State University Wexner Medical Center, Columbus, MI, United States; 4 LV Prasad Eye Institute, Hyderabad, India; 5 Orbital and Adnexal Service, Tiberia Hospital—GVM Care & Research, Rome, Italy; 6 Department of Translational Medicine, University of Ferrara, Ferrara, Italy; 7 Department of Ophthalmology, University of Naples Federico II, Napes, Italy

**Keywords:** clinical activity score (CAS), Graves’ ophthalmopathy, graves eye disease, orbital inflammation, orbital inflammatory disease, thyroid eye disease

Thyroid eye disease (TED) is an inflammatory and fibrotic orbitopathy most commonly associated with autoimmune thyroid dysfunction, particularly Graves disease. Its manifestations range widely, from mild irritation and tearing to significant proptosis, restrictive strabismus, exposure keratopathy, and compressive optic neuropathy. Although TED affects populations across the globe, its clinical expression is remarkably heterogeneous, shaped by genetic, ethnic, and geographic variables. Despite this global relevance, the field remains hindered by fragmented terminology, inconsistent diagnostic criteria, and significant variation in management strategies.

## The conundrum: TED remains trapped in a maze of many names and a lack of shared language

Recent advances in medical science have led to explosive growth in clinical research and drug development for TED, as well as advances in surgical techniques for rehabilitation. However, the study of this disease is significantly hampered by lack of consensus on terminology and basic guidelines for management. The disorder continues to be described with several overlapping names—Graves ophthalmopathy, Graves orbitopathy, endocrine ophthalmopathy, thyroid-associated orbitopathy and ophthalmopathy—reflecting the absence of a unified conceptual framework. Furthermore, terms such as “new onset” or “acute” might mean “within 6 months” or “within 12 months.” The term “chronic” might mean “stable, long-standing,” but there are no clear definitions of what stability or “long-standing” actually mean. The terminologic confusion is exemplified by the commonly used term “moderate-to-severe,” which just lumps together anything that is not “mild” or “vision-threatening.”

The lack of standardization also affects the clinical scoring systems used to assess TED. The NOSPECS classification emphasizes structural changes (1), whereas the Clinical Activity Score (CAS) focuses on inflammatory signs (2), and the VISA system evaluates visual function, inflammation, strabismus, and appearance (3). Even fundamental terminology such as *activity* is inconsistently defined across these frameworks and demonstrates substantial inter- and intra-observer variability. Particularly, the assessment of disease activity using CAS provides only a snapshot of a patient’s condition at a single visit, and has limited value for evaluating the broader clinical manifestations and progression of TED, reflecting its design origins in an era when corticosteroid therapy was the predominant treatment.

In addition, the orbit is a closed compartment prone to “compartment syndrome,” e.g. from obstruction of venous outflow, leading to congestion. Orbital congestion can be seen with vascular anomalies, orbital inflammation, orbital infection, and any other acute or sub-acute process that interferes with venous outflow. This can result in conjunctival chemosis and injection, orbital pressure and pain, caruncular edema, eyelid edema and erythema, and even dysmotility – all the same signs that currently comprise the CAS. Hence, CAS is not specific to inflammation and its items could be related to orbital congestion rather than true inflammatory activity. This mismatch can lead to conceptual misunderstanding and introduce bias in clinical trials, further complicating the interpretation of therapeutic outcomes.

## The consequences: without standardization, progress stalls and patients pay the price

In order for the field to progress, we need a clear understanding of terminology and a standardization across the globe that considers ethnic variations. Efforts by local Societies and regional organizations to establish TED guidelines often lead to further confusion, particularly since guideline development is often dominated by endocrinologists, with ophthalmologists underrepresented despite being the specialists most familiar with orbital anatomy, ocular motility, visual function, and surgical rehabilitation. The 2022 ATA/ETA consensus, for instance, included only two ophthalmologists among its nine authors (4), raising concerns about whether the nuanced clinical realities of TED were fully captured. Likewise, the European Group on Graves Orbitopathy (EUGOGO), an organization affiliated with the European Thyroid Association, has a membership structure that is theoretically balanced by requiring centers to have a combined clinic staffed by both an endocrinologist and an ophthalmologist. However, the number of oculoplastic specialists within this group remains limited, resulting in predominance of endocrinologists. Moreover, the EUGOGO thresholds for disease severity (5) do not fully reflect patient experience, particularly given the variability in functional and psychosocial impact. As a consequence of this longstanding ambiguity, several clinically important aspects of TED, such as ocular surface dysfunction and phenotypic variability, have been insufficiently represented or entirely omitted from many clinical trials. This lack of inclusion has contributed to variability in study methodology and, ultimately, to inconsistent or difficult-to-interpret outcomes.

Another example of the predominance of an endocrinology-oriented perspective in the TED literature is the limited citation of studies conducted by the International Thyroid Eye Disease Society (ITEDS), an international group composed primarily of ophthalmologists. Several ITEDS studies have generated essential data, including validated descriptions of inflammatory signs in TED and detailed analyses of intra-observer variability in proptosis measurement (6–8). Despite their methodological accurancy and multicenter design, these contributions have been only sparsely acknowledged in studies led by endocrinologists.

Collaboration with endocrinologists remains essential, but leadership in defining ocular disease must rest with those trained specifically in ophthalmology and oculoplastic surgery. Even with the best of intentions, placing primary responsibility for an eye-specific disorder in the hands of specialists without formal ophthalmic training can create gaps in perspective. Endocrinologists contribute essential expertise to the care of patients with TED, but their focus and training differ naturally from those of ophthalmologists. Collaborative efforts remain vital, yet clinical guidance is strongest when shaped by those whose expertise is most closely aligned with the structures and functions directly involved. Ensuring that ophthalmologists take a central role in defining terminology, diagnostic criteria, and management strategies allows recommendations to more accurately reflect the complexities of orbital disease and the visual system. This does not diminish the valuable contributions of other disciplines; rather, it ensures that multidisciplinary care is anchored in the expertise best positioned to lead it, ultimately improving consistency, safety, and outcomes for patients worldwide.

The critique of the guidelines currently in use further underscores the need for ophthalmology-driven standardization. It emphasizes the importance of foregrounding the quality-of-life impact of TED and calls for a clearer rationale behind the proposed shift from “inactive” to “stable” disease. Because clinicians have long relied on an active/inactive distinction, transitioning toward a framework emphasizing “progressive” versus “stable” disease demands explanation. Under this approach, the term “activity” would be reserved exclusively for progressive features, preventing confusion when determining treatment thresholds. Additional work will be required to develop guidelines for differentiating inflammatory versus congestive disease, since these entities require different treatment modalities.

## Moving forward

It is well past time for ophthalmologists to retake the lead on TED, based on our unique expertise. We now stand in a period of unprecedented therapeutic evolution for TED, with emerging and increasingly effective medical treatments reshaping clinical expectations and standards of care (9–14). Only an international ophthalmology-led group with expertise in orbital disease can provide proper clarity and consistency to the study and treatment of TED – a debilitating ophthalmic disorder. Such a group should be eager to collaborate with all stake holders, including endocrinologists, endocrine surgeons, radiation oncologists, nuclear medicine specialists and basic and clinical scientists, to achieve the best possible outcomes for patients worldwide. But decisions on the medical care of the eye, and formulation of the appropriate ophthalmic terminology, should be driven by ophthalmologists.

## References

[1] Werner SC . Modification of the classification of the eye changes of Graves’ disease: recommendations of the *Ad Hoc* Committee of the American Thyroid Association. J Clin Endocrinol Metab. (1977) 44:203–4. doi: 10.1210/jcem-44-1-203, PMID: 576230

[2] Mourits MP Koornneef L Wiersinga WM Prummel MF Berghout A van der Gaag R . Clinical criteria for the assessment of disease activity in Graves’ ophthalmopathy: a novel approach. Br J Ophthalmol. (1989) 73:639–44. doi: 10.1136/bjo.73.8.639, PMID: 2765444 PMC1041835

[3] Dolman PJ . Grading severity and activity in thyroid eye disease. Ophthalmic Plast Reconstr Surg. (2018) 34:S34–40. doi: 10.1097/IOP.0000000000001150, PMID: 29952931

[4] Ross DS Burch HB Cooper DS Greenlee CM Laurberg P Maia AL . 2022 American thyroid association and European thyroid association guidelines for the management of thyroid eye disease. Thyroid. (2022) 32:278–313. doi: 10.1089/thy.2021.0135

[5] Bartalena L Kahaly GJ Baldeschi L Dayan CM Eckstein A Marcocci C . The 2021 European Group on Graves’ orbitopathy (EUGOGO) clinical practice guidelines for the medical management of Graves’ orbitopathy. Eur J Endocrinol. (2021) 185:G43–67. doi: 10.1530/EJE-21-0479, PMID: 34297684

[6] Bingham CM Sivak-Callcott JA Gurka MJ Nguyen J Hogg JP Feldon SE . Axial globe position measurement: A prospective multicenter study by the international thyroid eye disease society. Ophthalmic Plast Reconstr Surg. (2016) 32:106–12. doi: 10.1097/IOP.0000000000000437, PMID: 25719380 PMC4549213

[7] Dolman PJ Cahill K Czyz CN Douglas RS Elner VM Feldon S . Reliability of estimating ductions in thyroid eye disease: an International Thyroid Eye Disease Society multicenter study. Ophthalmology. (2012) 119:382–9. doi: 10.1016/j.ophtha.2011.07.011, PMID: 21959369

[8] Mawn LA Dolman PJ Kazim M Strianese D Genol I Chong KKL . Soft tissue metrics in thyroid eye disease: an international thyroid eye disease society reliability study. Ophthalmic Plast Reconstr Surg. (2018) 34:544–6. doi: 10.1097/IOP.0000000000001080, PMID: 29465482

[9] Strianese D . Update on Graves disease: advances in treatment of mild, moderate and severe thyroid eye disease. Curr Opin Ophthalmol. (2017) 28:505–13. doi: 10.1097/ICU.0000000000000402, PMID: 28700384

[10] Barbesino G Salvi M Freitag SK . Future projections in thyroid eye disease. J Clin Endocrinol Metab. (2022) 107:S47–56. doi: 10.1210/clinem/dgac252, PMID: 36346684 PMC9359449

[11] Subramanian PS Cho RI Kahana A . Efficacy of teprotumumab therapy in patients with long-duration thyroid eye disease. Curr Opin Ophthalmol. (2023) 34:487–92. doi: 10.1097/ICU.0000000000000997, PMID: 37610428

[12] Kahaly GJ Subramanian PS Conrad E Holt RJ Smith TJ . Long-term efficacy of teprotumumab in thyroid eye disease: follow-up outcomes in three clinical trials. Thyroid. (2024) 34:880–9. doi: 10.1089/thy.2023.0656, PMID: 38824618

[13] Ciarmatori N Quaranta Leoni F Quaranta Leoni FM . Redefining treatment paradigms in thyroid eye disease: current and future therapeutic strategies. J Clin Med. (2025) 14:5528. doi: 10.3390/jcm14155528, PMID: 40807149 PMC12347536

[14] Selva D Lane CM Mordekar SR . Re-activation of thyroid eye disease. Clin Exp Ophthalmol. (2004) 32:46–50. doi: 10.1111/j.1442-9071.2004.00747.x 14746591

